# Characterization of Agronomic and Seed Oil Features for Different Cultivars of Tree Peony

**DOI:** 10.3390/plants12173112

**Published:** 2023-08-30

**Authors:** Hao Wang, Shuo Wei, Yinglong He, Xiaohui Wang, Yuying Li, Dongfeng Wei, Zhanying Wang, Lili Guo, Muhammad Shaaban, Xiaogai Hou

**Affiliations:** 1 College of Agriculture, Henan University of Science and Technology, Luoyang 471000, China; wanghao981209@163.com (H.W.); hnzywei@163.com (S.W.); hyl8282.good@163.com (Y.H.); lixueer593@126.com (Y.L.); shabanbzu@hotmail.com (M.S.); 2 Luoyang Academy of Agricultural and Forestry Sciences, Luoyang 471000, China; wxhnky@126.com (X.W.); lnhhzx@126.com (Z.W.); 3 Luoyang Vocational and Technical College, Luoyang 471000, China; weidongfeng000@163.com

**Keywords:** agronomic characters, cultivar screening, grey relational analysis, oil properties, oil tree peony

## Abstract

Tree peony is a unique oil plant resource in China, and tree peony seed oil is one of the healthy edible oils with a very promising future. However, the main oil tree peony cultivars promoted in China are *Paeonia ostii* ‘Fengdan’ and *Paeonia rockii*. In order to explore new oil tree peony cultivars, 68 tree peony cultivars were investigated and cultivars with oil potential were selected by cluster analysis and grey relational analysis (GRA) in this study. The results demonstrated that the 68 cultivars varied significantly in terms of agronomic characteristics (*p* < 0.05), with the coefficient of variation in seed yield per plant reaching a high of 75.36%. The oil content of 46 cultivars was higher than ‘Fengdan’ (20.87 ± 0.26%) and ‘Zibanbai’ (21.24 ± 1.01%), while the alpha-linolenic acids and total unsaturated fatty acid contents of 26 cultivars were higher than ‘Fengdan’ (39.79 ± 1.13% and 88.99 ± 0.47%) and ‘Zibanbai’ (40.51 ± 0.09% and 93.59 ± 0.09%). Finally, three cultivars with better integrated traits were selected by cluster analysis and grey relational analysis (GRA), comprising of ‘Changshoule’, ‘Xianchizhenghui’, and ‘Yupantuojin’. The contents of alpha-linolenic acids and total unsaturated fatty acids in ‘Changshoule’ (47.98 ± 0.17% and 93.60 ± 0.08%), ‘Xianchizhenghui’ (49.44 ± 0.63% and 93.80 ± 0.06%), and ‘Yupantuojin’ (40.46 ± 0.26% and 93.58 ± 0.06%) were higher than that of ‘Fengdan’ (39.79 ± 1.13% and 88.99 ± 0.47%). In general, these cultivars can be used as hybrid parental materials for breeding new excellent oil tree peony cultivars.

## 1. Introduction

Tree peony (*Paeonia* section *Moutan* DC.) is a perennial shrub native to China and has been widely introduced around the world for over 2000 years [1]. It is well known for its large and colorful flowers with high ornamental, medicinal, and economic value. In recent years, the oil value of tree peony seeds has attracted increasing attention and acceptance [2,3]. Tree peony seed oil is rich in unsaturated fatty acids (UFAs), fat-soluble vitamin E, and squalene. These compounds are important for improving dietary structure and human health [3]. Thus, the tree peony has become a new alternative source of edible oil and is being grown on a large scale in China.

Polyunsaturated fatty acids (PUFAs) are mainly classified into n-3 and n-6 fatty acids. Studies have shown that alpha-linolenic acids (n-3) and linoleic acid (n-6) are essential for humans because they cannot synthesize these two PUFAs, meaning they must be obtained from dietary sources [4]. Humans consume PUFAs mostly through vegetable oils, and each type of vegetable oil has a unique PUFA composition and content that offers the body a variety of nutrients [5,6,7]. The PUFA composition and levels in edible oils play an essential role in human health, particularly the n-3 to n-6 FA ratio [8,9]. However, the content of n-6 PUFAs in modern human dietary oils is generally high, while n-3 PUFAs are generally deficient [10]; alpha-linolenic acids are less than 10% in most popular edible oils such as sunflower oil, peanut oil, palm oil, corn oil, soybean oil, rapeseed oil, and olive oil [11]. Tree peony oil is a good source of edible oil with a high content of alpha-linolenic acids (>40%). However, the current shortage of oil tree peony cultivars is an important factor restricting the development of the industry.

Research on the oil value of tree peony seeds focuses on oil extraction, processing, and fatty acid composition analysis. However, little research pays attention to the screening and evaluation of oil-using cultivars. Moreover, the available research has several limitations: 1. There is an insufficient number of cultivars in the trials, meaning the sample sizes are small [12,13,14]. 2. The screening methods are not sufficiently scientific and systematic. Seed yield is the primary consideration in terms of production, and it is directly related to the final oil yield [15,16]. However, seed yield-related agronomic traits have not been comprehensively characterized in previous studies [17].

In response to these issues, this study conducted the following work: (1) Based on the progress of the previous research group, 68 tree peony cultivars were selected based on characteristics of seed yield per plant, seed number, number of fruits per plant, seed length, seed width, and hundred seed weight (HSW) to investigate the effect of each trait on yield. (2) The oil properties including kernel contents, oil contents, and fatty acid composition of 68 cultivars were measured. (3) The cultivars suitable for oil cultivation were selected by cluster analysis and grey relational analysis (GRA). The main objective of this study is to screen out cultivars with potential oil utilisation, which can further promote the development of the tree peony seed oil industry and the development of health food using tree peony seed oil as the main raw material.

## 2. Materials and Methods

### 2.1. Plant Material 

The tree peony cultivars (Table A1) utilized in this study were planted in the tree peony cultivation experimental base of Henan University of Science and Technology (112°25′40.29″ E, 34°36′6.47″ N), which has a warm temperate continental monsoon climate with four distinct seasons and simultaneous rain and heat. The main oil tree peony cultivars used for promotion at present, ‘Fengdan’ (one of the cultispecies of *Paeonia ostii* ‘Fengdan’) and ‘Zibanbai’ (one of the cultispecies of *Paeonia rockii*), were selected as the control for new varieties screening. Approximately 120 g of seed was selected from each cultivar, dried to a consistent weight using an electrically heated blast dry box (DHG-9070A, Shanghai, China) at 110 °C, and then stored in a nitrogen-filled desiccator. Independent samples were analysed in triplicate. 

### 2.2. The Agronomic Traits of Tree Peony

The seed yield per plant (Y) refers to the dry weight of whole plant seeds. The seed number per plant and the number of fruits per plant refer to the number of seeds and fruits of the whole plant, respectively. The description of the measurement method for HSW can be found in the reference literature [18,19]. The width of the seed is the maximum measured value perpendicular to the extension direction of the seed stalk, while the seed length is the opposite. The values were measured using vernier calipers with an accuracy of 0.01 mm [17]. 

### 2.3. The Kernel and Oil Contents in the Seeds of Different Tree Peony Cultivars

The dried tree peony seeds were shelled and separated to get the kernels. The kernel contents were the weight percentage of the kernel and seed [20]. Tree peony seed oil was extracted using a supercritical carbon dioxide (CO_2_) extraction device (HA220-50-06, Nantong, China). The extraction temperature of the supercritical CO_2_ extractor was set at 45 °C, the extraction pressure at 30 MPa, the extraction time at 2.8 h, the separation temperature at 35 °C, and the separation pressure at 11.5 MPa. The calculation of oil content is described in the literature [21,22].

### 2.4. Fatty Acid Methylation

A previously described methylation method was applied [23,24]. After being previously prepared, the seed oil was redissolved in 1.0 mL of a methanol solution containing 5% concentrated sulfuric acid, vortexed for 1 min, and then heated in a water bath at 60 °C for 1.5 h to induce derivatization. Afterward, the samples were cooled to room temperature. The reaction was then terminated by adding 2.0 mL of water. Fatty acid methyl ester (FAME) was subsequently extracted with 2.0 mL of n-pentane. After centrifugation at 3500 rpm for 10 min, the supernatant was collected and dried using a nitrogen blowing device. The concentrated solution was redissolved in an appropriate volume of n-hexane and transferred to the injection vial for GC–MS before being transferred to a 2 mL injection vial. Moreover, 20 μL of tridecanoic acid methyl ester (10.0 g·L^−1^ in n-hexane) was utilized for this investigation as an internal standard.

### 2.5. Gas Chromatograph–Mass Analysis

The fatty acids (FAs) composition and content of tree peony seed oil were measured by the method described in the literature [25,26]. FA analysis was carried out using a gas chromatograph–mass spectrometer (7890A-5975, Agilent Technologies Inc., Santa Clara, CA, USA). The column was an HP-5MS (Agilent, USA) fused silica capillary column. The operating conditions are listed below: 0.8 mL·min^−1^ of ultra high-quality helium was employed as the carrier gas, and the analysis was carried out in constant flow mode. The injector temperature was set at 250 °C for split injection with a split ratio of 30:1, while the transfer line, ion source, and quadrupole temperatures were set at 280, 230, and 150 °C, respectively. 1 μL was the injection volume. The oven’s initial temperature of 80 °C was maintained for 2 min, after which it was raised to 230 °C by 10 °C min·L^−1^ and remained isothermal for 5 min. The mass-selective detector’s ionization potential was 70 eV, and its scan range was 30–450 amu. Triplicate analyses were out. The mass spectra database search (NIST17 Library) was used to identify the compounds.

### 2.6. Grey Relational Analysis

Firstly, cluster analysis was conducted on the Y of 68 cultivars, and the cultivars with higher yields were pre-selected. The measurement standard of the clustering method used Euclidean distance. Secondly, an analytical evaluation of the higher yielding tree peony cultivars was carried out using GRA [27,28]. Taking higher yielding tree peony cultivars as a grey system and each candidate cultivar as a factor of the system, we calculated the correlation degree between the factors and evaluated the similarity between the determinants according to the correlation degree. First, a reference cultivar is set up and a reference sequence, X_0_, is composed of each character index of the reference cultivars. Since the screening objectives of this study are positive indicators (i.e., the larger the better), the maximum value of each trait in this study can be selected into the reference sequence, X_0_, as the optimal value. The candidate cultivars constitute the comparison sequence, X_i_. As the units of measurement for each trait are different, the raw data needed to be dimensionless. This study involved a comprehensive evaluation of multiple indicators, so the extreme value method was used, i.e., the data for each sequence was divided by the corresponding X_0_ value. The calculation formula of GRA is as follows:

(1) $$\xi_{i}\left(K_{j}\right)=\frac{\underset{i}{\min}\underset{j}{\min}\Delta i\left(K_{j}\right)+\rho \underset{i}{\max}\underset{j}{\max}\Delta i\left(K_{j}\right)}{\Delta i\left(K_{j}\right)+\rho \underset{i}{\max}\underset{j}{\max}\Delta i\left(K_{j}\right)}$$

(2) $$\gamma_{i}=\frac{1}{n}\overset{n}{\underset{j=1}{\sum }}\xi_{i}\left(K_{j}\right)$$

ρ was a distinguishing coefficient, which can be set from 0 to 1. Setting different ρ values would lead to different GRA values while the trend and order of GRA series will not be changed. In this work, ρ was set as 0.5, as has been previously reported [29]. n is the number of GRA values, i is the trial number, and j ranges from 0 to 11. $\Delta i\left(K_{j}\right)$ was calculated as:

(3) $$\Delta i\left(K_{j}\right)=\left|X_{0}\left(K_{j}\right)-X_{i}\left(K_{i}\right)\right|$$

## 3. Results and Discussion

### 3.1. Agronomic Traits of Different Tree Peony Cultivars

This study found that the seed yield per plant (Y) of different tree peony cultivars varied greatly, ranging from 31.22 g to 337.22 g per plant, with a coefficient of variation (CV) as high as 75.36% and a mean value of 62.75 g (Table 1, Table A2). Among all the investigated tree peony cultivars, the Y of No.46 (‘Zibanbai’) was the highest, reaching 337.22 g, and was followed by No.39 (‘Yupantuojin’), reaching 143.56 g. There were five cultivars with a Y of more than 100 g, namely, No.62 (‘Changshoule’), No.33 (‘Xianchizhenghui’), No.22 (‘Caiyelanju’), No.36 (‘Xueyuanhongxing’), and No.38 (‘Ruhuasiyu’). The Y of ‘Fengdan’ is similar to that of previous studies [30]. The CV of the number of seeds was 61.92%, and No.46 (‘Zibanbai’) had the highest number of seeds among the investigated cultivars, with more than 900, followed by No.33 (‘Xianchizhenghui’). The cultivars with a Y of more than 100 g also had a high seed number. Most of these cultivars have more than 300 seeds, which implies a high correlation between seed number and Y. The levels of Y were consistent with those reported in the literature [18]. Overall, the highest CV was found for Y (75.36%), followed by seed number (61.92%), number of fruits per plant (43.75%), hundred seed weight (19.27%), seed length (10.24%), and seed width (8.56%). 

The greater the CV, the more pronounced the differences between cultivars. In this study, the CV of different agronomic traits, ranging from 8.65% to 75.36%, indicates that the 68 tree peony cultivars exhibit a rich genetic diversity and different phenotypic traits vary widely (Table 1, Table A2). Of these traits, the highest CV value was the Y (75.36%), indicating that there are great differences between the Y of different tree peony cultivars; this situation is conducive to the selection and evaluation of superior plants [31]. The yield could directly affect the final oil content and was a good screening index to evaluate the oil potential of different cultivars [18].

**Figure 1:**
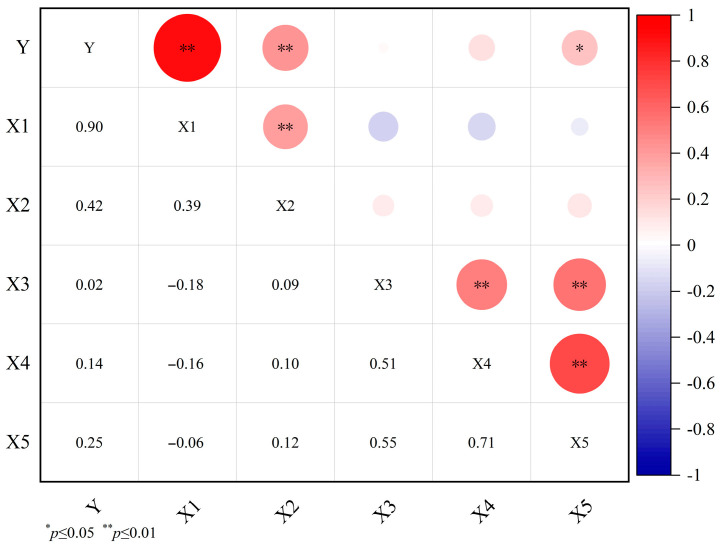
Correlation analysis of seed yield per plant and yield component traits. Red indicates a positive correlation and blue indicates a negative correlation. The color depth and the circle area represent the size of the correlation coefficient.

To investigate the effect of different traits on Y, the correlations between Y and other traits were analyzed (Figure 1). The Y significantly and positively correlated with the seed number (X1) and the number of fruits per plant (X2). The seed number had the strongest correlation with Y (*r* = 0.90, *p* < 0.01), followed by the number of fruits per plant (*r* = 0.42, *p* < 0.01). The results showed that the seed number and the number of fruits per plant are the main factors influencing the Y. These two agronomic traits could be used as indicators to evaluate Y and may play important roles in selective breeding for high yield [31,32,33,34,35]. Previous studies have not explored the Y components of oil tree peonies in depth and most have focused on oil traits [12,20,36]. However, systematic research on the Y formation is the theoretical basis for achieving high-yield breeding and should be given high priority [37]. In our studies, the relationship between the various characteristics of 68 tree peony cultivars were analyzed and the main agronomic traits were clarified. These results provide important guiding context for the reasonable evaluation and exploration of oil tree peony germplasm resources, scientific cultivation and management, and improving breeding efficiency [35].

### 3.2. The Kernel and Oil Contents of Different Tree Peonies

**Figure 2:**
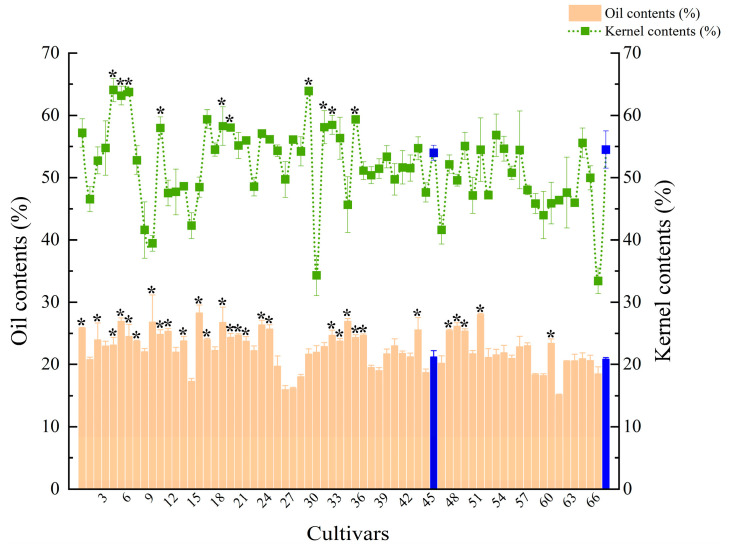
Kernel content and oil content of different tree peony cultivars. Blue indicates the ‘Fengdan’ and ‘Zibanbai’ cultivars. * indicates that the value is significantly greater than ‘Fengdan’ and ‘Zibanbai’ (*p* < 0.05).

This experiment measured the kernel and oil contents of 68 cultivars (Figure 2). The kernel content varied significantly, from 33.41 ± 2.05% to 64.06 ± 1.89%, with No.5 (‘Sihonglian’) having the highest kernel content. The kernel contents of nine cultivars were significantly higher than No.68 (‘Fengdan’) (54.50 ± 2.96%) (*p* < 0.05). This value represents the degree of fullness of the seed and is correlated with habitat factors such as altitude, latitude, average annual temperature, and soil N, P, and K contents [33,38]. The oil content ranged from 15.13 ± 0.14%~28.25 ± 1.30%, and the highest was found in No.16 (‘Xiaotaohong’), followed by No.52 (‘Hepingfen’) at 27.99 ± 0.20%; the lowest was found in No.62 (‘Changshoule’), at only 15.13 ± 0.14%. The oil content of 29 cultivars was significantly higher than ‘Fengdan’ (20.87 ± 0.26%) and ‘Zibanbai’ (21.24 ± 1.00%) (*p* < 0.05). Most of the cultivars have oil content of more than 20%, which is consistent with the literature [39,40]. This shows that other tree peony cultivars also have the potential for oil use. Kernel and oil content are important traits for oil use and their differences are related to many factors such as cultivar differences, crop management, water and fertilizer application, etc. [41,42,43].

### 3.3. Composition and Content of FAs among Different Cultivars 

The FA content of tree peony was mainly dominated by palmitic acid (PA), stearic acid (SA), oleic acid (OA), linoleic acid (LA), and alpha-linolenic acids (ALA) [44,45,46,47,48]. These main five fatty acid contents of 68 cultivars were analyzed by GC–MS (Table 2). The content of ALA ranges from 28.02 ± 0.60% to 53.02 ± 0.46%, with an average content of 41.41 ± 5.40%, which is the highest of the five dominant FAs. Two samples, No.4 (‘Sijinpaohong’) and No.28 (‘Baihuazhancui’), were notable for high ALA content among all samples (53.02 ± 0.46% and 52.55 ± 0.12%, respectively), while No.1 (‘Jinguimantang’) had the lowest ALA content. LA and OA were also two important FAs with higher contents, which were 19.24 ± 0.20%~37.74 ± 0.17% and 16.21 ± 0.82%~29.82 ± 0.29%, respectively. Sample No.1 (‘Jinguimantang’) had the highest LA content (37.74 ± 0.17%), while sample No.4 (‘Sijinpaohong’) had the lowest (19.24 ± 0.20%). Sample No.64 (‘Hongguishizi’) had the highest OA level (29.82 ± 0.29%), while sample No.25 (‘Jianshifen’) had the lowest content (16.21 ± 0.82%). Most samples indicated low levels of two saturated fatty acids (PA and SA). The PA and SA contents were, respectively, 3.43 ± 0.09% to 7.14 ± 0.24% and 0.31 ± 0.02% to 2.31 ± 0.06%. The TUFA content ranged from 88.99 ± 0.47% to 95.26 ± 0.25%. The TUFA content of No.29 (‘Qinglongyaojinmo’) was the highest while No.68 (‘Fengdan’) had the lowest. This study revealed that, compared to various typical edible oils such as olive oil, rapeseed oil and soybean oil, tree peony seed oil had higher quantities of ALA and TUFA [25]. Most cultivars had higher ALA and TUFA concentrations than the oil tree peony ‘Fengdan’, which has outstanding nutritional and economic value to be developed into an oil tree peony.

**Figure 3:**
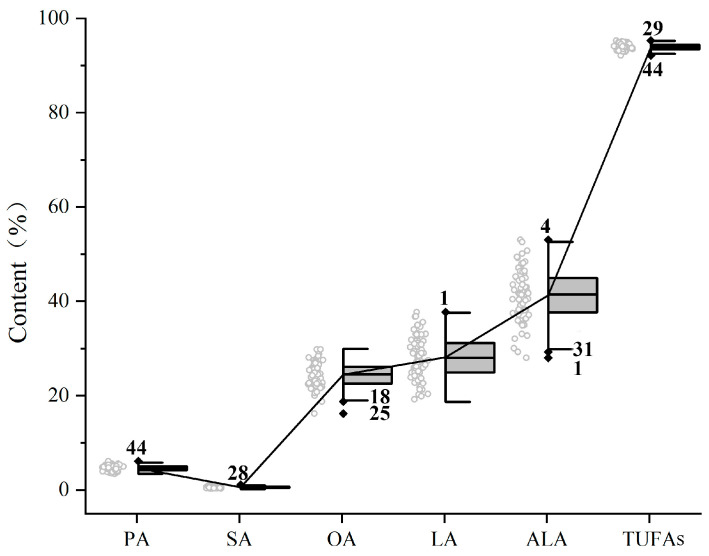
The range and distribution of five major FAs and total FA content in 68 tree peony cultivars.

**Figure 4:**
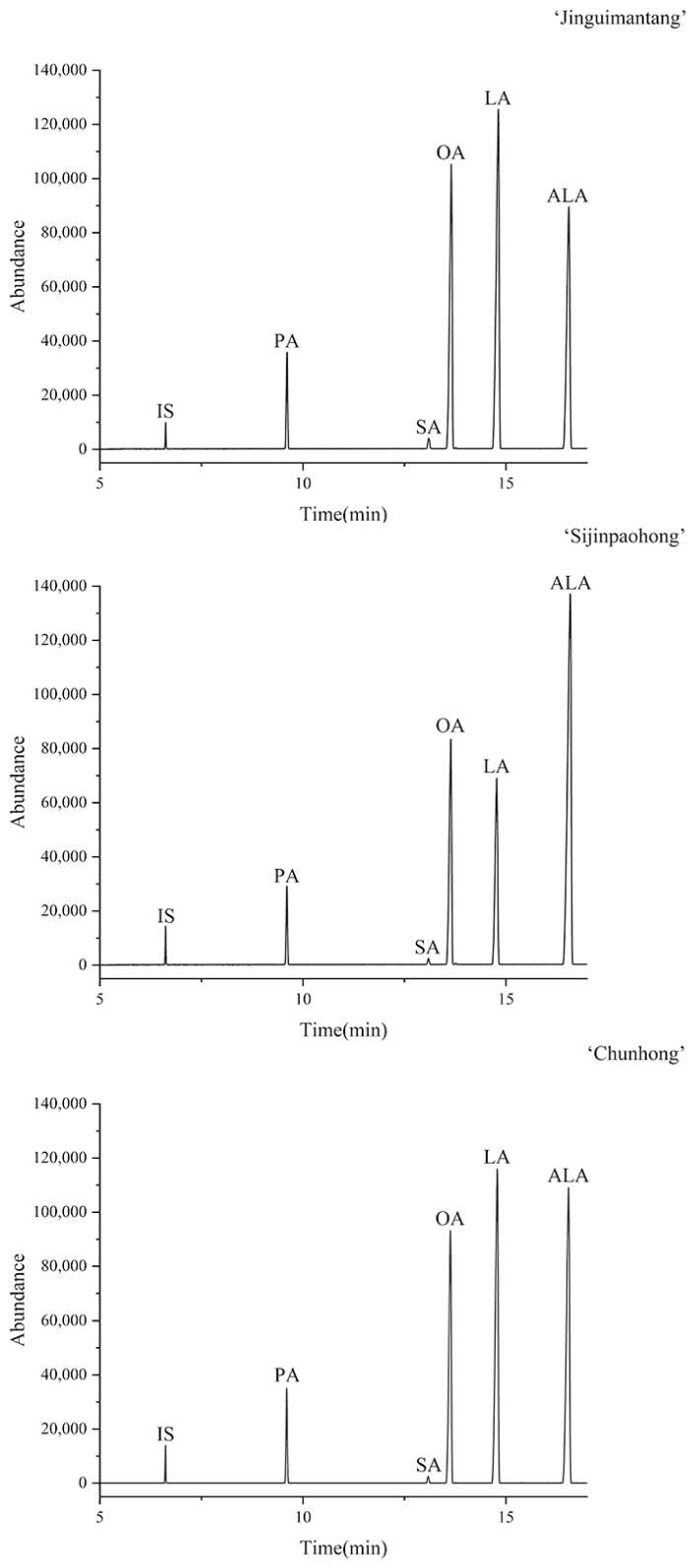
GC–MS chromatograms of fatty acid extracted from seeds of three representative cultivars. Peaks: IS = C13:0 (internal standard), PA = C16:0 (palmitic acid), SA = C18:0 (stearic acid), OA = C18:1Δ9c (oleic acid), LA = C18:2Δ9c, 12c (linoleic acid), ALA = C18:3Δ9c, 12c, 15c (α-linolenic acid).

The distribution of the 68 cultivars’ five major fatty acid (PA, SA, OA, LA and ALA) and TUFA contents was also investigated in this study (Figure 3). The predominant UFAs of most cultivars were ALA, followed by LA and OA, with a few exceptions, such as samples No.1 (‘Jinguimantang’), No.4 (‘Sijinpaohong’), and No.12 (‘Chunhong’). The former was dominated by LA, followed by OA and ALA (Figure 4). Sample No.4 (‘Sijinpaohong’) had the highest content of ALA, followed by OA and LA. No.12 (‘Chunhong’) had a small difference between its LA and ALA contents. As a result, the three cultivars mentioned above may be ideal reference materials for studying the biosynthesis of ALA in tree peony seeds. In addition, No.29 (‘Qinglongyaojinmo’) had significantly a higher TUFA content than the other cultivars, reaching 95.26 ± 0.25% (*p* < 0.001). The 28 cultivars’ OA, LA, and ALA contents complied with the requirements of the industry standard LS/T3242-2014 (Food Industry Standard for Peony Seed Oil of the People’s Republic of China). The levels of TUFAs were consistent with previous studies [20,40]. As the composition of tree peony seed oil is highly similar, different cultivars of tree peony seeds can be mixed to extract the oil, facilitating industrial mass production. Screening out high-quality and high-yield tree peony cultivars can bring considerable economic benefits.

The amount of unsaturated fatty acids in the daily diet profoundly affects human health, and many disorders are associated with low ratios of n-3/n-6 PUFAs [14,49]. According to the Food and Agriculture Organization of the United Nations (FAO) and the World Health Organization (WHO), the recommended dietary n-3/n-6 PUFAs ratio is higher than 1/5 [50]. In this experiment the mean value of n-3/n-6 ratio of 68 cultivars was 1.55, with the ratio of 1.55 and 1.56 for *P. ostii* ‘Fengdan’ and ‘Zibanbai’, respectively. The highest was found in No.4 (‘Sijinpaohong’), reaching 2.76, which is 13.80 times the recommended intake ratio and more than 1.7 times the ratio of *P. ostii* ‘Fengdan’ and ‘Zibanbai’. This means that No.4 (‘Sijinpaohong’) can be used as a breeder for the cultivars that are high in the ratio of n-3/n-6 PUFAs. The lowest of the 68 cultivars, No.1 (‘Jinguimantang’), also reached 0.75, which is 3.75 times the recommended intake ratio, making tree peony seed oil an important resource for restoring balance to dietary n-3/n-6 ratios. Tree peony seed oil also has promise as a n-3 PUFAs supplement. Therefore, in the subsequent screening of cultivars, ALA and TUFAs were used as the main indicators to further evaluate the quality of different cultivars of tree peony seed oil.

### 3.4. Screening of High Yielding and High Quality Tree Peony Cultivars

**Figure 5:**
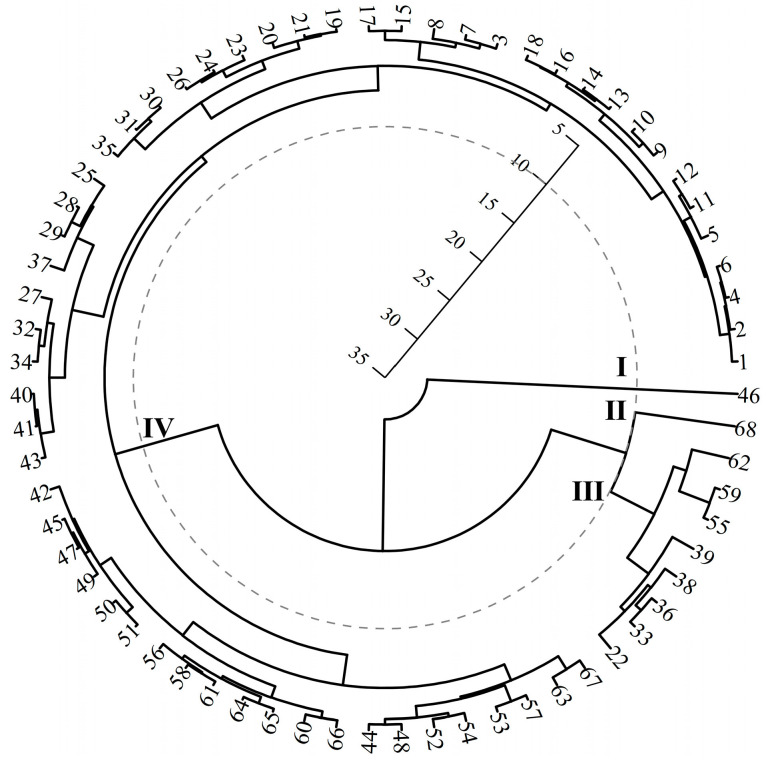
The results of cluster analysis on 68 tree peony cultivars.

In order to comprehensively assess the agronomic traits and oil properties of different cultivars, this study first carried out a cluster analysis based on the Y of the 68 tree peony cultivars. Results showed that the participating cultivars could be divided into four categories at a squared Euclidean distance of 10 (Figure 5). Classes I and II are No.46 (‘Zibanbai’) and No.68 (‘Fengdan’), respectively, with average Y of 337.22 g and 196.30 g. Class III consists of ten cultivars, including No.55 (‘Mudanyan’), No.59 (‘Daonaiteng’), No.62 (‘Changshoule’), and so on, whose average Y was 122.61 g. Class IV consists of 58 cultivars and its average Y was 47.45 g. The Y of first three categories of cultivars ranged from 97.27 g to 337.22 g and had an average value of 151.44 g, which was significantly higher than that of the Class IV.

**Figure 6:**
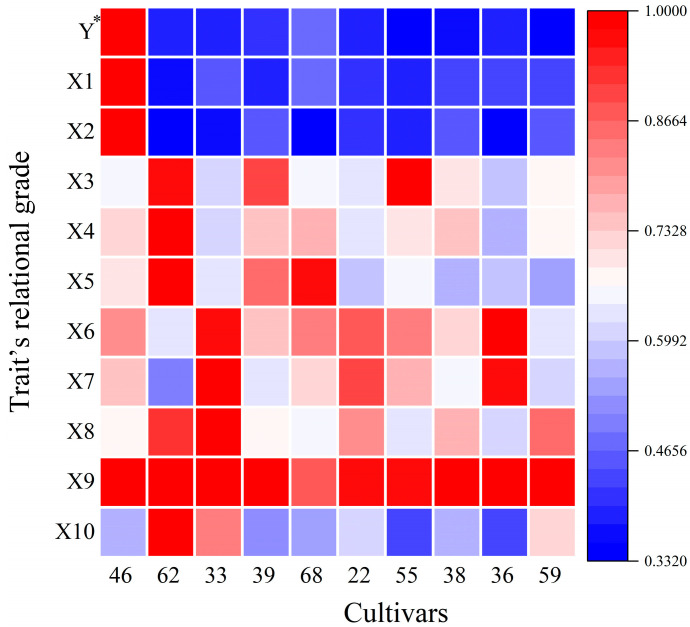
The traits’ relational grade of high yield tree peony cultivars. * Seed yield per plant (Y), Seed number per plant (X1), Number of fruits per plant (X2), Seed length (X3), Seed width (X4), Hundred seed weight (X5), Kernel contents (X6), Oil contents (X7), ALA content (X8), Total UFA content (X9), n-3/n-6 (X10).

GRA is an effective method to analyze multiple factors [29,51]. It can quantitatively evaluate the main characteristics of the test object and overcome the limitations of pairwise comparison of single characters [52,53,54]. Given that there are eleven traits in this study, this study used GRA for further systematic selection of the first three categories. In this study, the reference cultivar was assumed, according to grey system theory, to basically cover the target traits required for oil tree peonies. According to the principle of GRA, the greater the correlation of a cultivar, the closer the cultivar is to the reference cultivar, and by comparing the correlation of different traits of different cultivars, the advantages and disadvantages of the cultivar can be clarified [51]. According to the GRA results (Figure 6), ‘Zibanbai’ is in first place; it has been popularly grown as an oil cultivar in some parts of China [55,56,57] and also illustrates the reliability of the screening method in this study. There were three cultivars with a higher correlation than No.68 (‘Fengdan’), namely, No.62 (‘Changshoule’), No.33 (‘Xianchizhenghui’), and No.39 (‘Yupantuojin’). These three cultivars have potential value for oil promotion.

‘Changshoule’ (No. 62) had a relation coefficient of 0.7375 and belongs to the Japanese tree peony cultivar group. It had the highest HSW, seed width, and n-3/n-6 ratio of the 10 cultivars (29.33 ± 0.33 g, 9.47 ± 1.11 mm and 2.25, respectively) (Table 3). In terms of ALA content, TUFA content, and n-3/n-6 ratio, it is superior to No.68 (‘Fengdan’) and ‘Zibanbai’ (No. 46) and ranks among the top 10 cultivars. These traits are important indicators when screening oil use cultivars [20,23]. The third highest correlation was for ‘Xianchizhenghui’ (No.33) at 0.7101, which had the highest oil content and ALA content at 24.69 ± 0.82% and 49.44 ± 0.63%, respectively. Excellent performance was seen in oil use traits such as kernel content, oil content, ALA content, TUFA content, and n-3/n-6 ratio, all of which were higher than in No.68 (‘Fengdan’), indicating high development potential. The fourth was ‘Yupantuojin’ (No.39), which belongs to the Zhongyuan tree peony cultivar group, with a relation coefficient of 0.6633. Its seed length, HSW, and TUFA content performed well, at 11.00 ± 0.61 mm, 27.36 ± 0.45 g, and 93.58 ± 0.06%, respectively, but the performance of other traits was average. Using the GRA method, three cultivars with good performance in traits closely related to economic efficiency, such as seed yield per plant, oil content, and fatty acid content, were finally obtained, indicating that they can be applied in practical production [20,23,58]. Due to its high nutritional value, tree peony seed oil has a huge market requirement, and the related industry has developed rapidly in recent years [23,38]. However, the lack of suitable cultivars is a major constraint to the development of the industry. The excellent cultivars screened in this study may be good candidates as raw material for edible oils and could alleviate the current situation. 

## 4. Conclusions

In this study, 68 tree peony cultivars were evaluated in order to select those with potential for oil use. Based on agronomic traits and seed oil properties, a total of three cultivars including ‘Changshoule’, ‘Xianchizhenghui’, and ‘Yupantuojin’ were recognized as potential candidates for oil-use by cluster analysis and GRA. With a good combination of traits, these cultivars are good resources for tree peony seed oil production and would contribute to the development of tree peony as a woody oil plant and industrial crop for research and development applications. 

## Figures and Tables

**Table 1:** The variation analysis of seed yield and yield components of 68 tree peony cultivars.

Index	Y (g) *	X1	X2	X3 (mm)	X4 (mm)	X5 (g)
‘Fengdan’	196.30	524.67	23.00	9.13	8.33	28.81
‘Zibanbai’	337.22	907.67	100.33	9.20	8.13	24.65
Min	31.22	83.33	13.00	7.40	5.83	12.93
Max	337.22	907.67	100.33	11.50	9.47	29.33
Mean	62.75	225.75	37.39	9.37	7.65	21.43
SD	47.29	139.78	16.36	0.96	0.66	4.13
CV	75.36%	61.92%	43.75%	10.24%	8.65%	19.27%

* Y: Seed yield per plant, X1: Seed number per plant, X2: Number of fruits per plant, X3: Seed length, X4: Seed width, X5: Hundred seed weight.

**Table 2:** The FA content of the investigated samples (mean ± SD, n = 3).

NO.	PA (%) *	SA (%)	OA (%)	LA (%)	ALA (%)	TUFA (%)	n-3/n-6
**1**	**4.82 ± 0.21 ^&^**	**0.68 ± 0.09 ^&^**	**27.70 ± 0.58 ^&^**	**37.74 ± 0.17 ^&^**	**28.02 ± 0.59 ^&^**	**93.45 ± 0.24 ^&^**	**0.74**
2	4.96 ± 0.75 ^&^	0.42 ± 0.03 ^&^	25.93 ± 0.26 ^&^	30.29 ± 2.70 ^&^	37.46 ± 3.52 ^&^	93.68 ± 0.87 ^&^	1.24
3	4.85 ± 0.03 ^&^	0.65 ± 0.02 ^&^	26.73 ± 0.13 ^&^	23.92 ± 0.04 ^&^	42.97 ± 0.16 ^&^	93.62 ± 0.08 ^&^	1.80
**4**	**4.29 ± 0.06 ^&^**	**0.41 ± 0.01 ^&^**	**22.22 ± 0.26 ^&^**	**19.24 ± 0.20 ^&^**	**53.02 ± 0.46 ^&^**	**94.48 ± 0.30 ^&^**	**2.76**
5	4.54 ± 0.20 ^&^	0.94 ± 0.05 ^&^	23.39 ± 0.03	21.76 ± 0.06 ^&^	48.39 ± 0.10 ^&^	93.54 ± 0.13 ^&^	2.22
6	3.72 ± 0.08 ^&^	0.61 ± 0.03 ^&^	27.55 ± 0.09 ^&^	25.94 ± 0.12	41.23 ± 0.22	94.72 ± 0.08 ^&^	1.59
7	5.35 ± 0.02 ^&^	0.96 ± 0.01 ^&^	23.83 ± 0.20	33.05 ± 0.10 ^&^	36.06 ± 0.52 ^&^	92.94 ± 0.26 ^&^	1.09
8	3.71 ± 0.09 ^&^	0.42 ± 0.08 ^&^	22.52 ± 0.31 ^&^	34.80 ± 0.04 ^&^	37.65 ± 0.37 ^&^	94.97 ± 0.04 ^&^	1.08
9	3.87 ± 0.06 ^&^	0.39 ± 0.03 ^&^	24.85 ± 0.12 ^&^	31.87 ± 0.05 ^&^	38.16 ± 0.04	94.88 ± 0.07 ^&^	1.20
10	5.30 ± 0.06 ^&^	0.67 ± 0.03 ^&^	25.32 ± 0.03 ^&^	24.89 ± 0.05	42.95 ± 0.15 ^&^	93.16 ± 0.14 ^&^	1.73
11	4.79 ± 0.08 ^&^	0.42 ± 0.04 ^&^	23.66 ± 0.08	34.63 ± 0.14 ^&^	35.47 ± 0.04 ^&^	93.76 ± 0.12 ^&^	1.02
12	4.39 ± 0.02 ^&^	0.36 ± 0.01 ^&^	23.31 ± 0.17	31.16 ± 0.06 ^&^	40.01 ± 0.30	94.48 ± 0.08 ^&^	1.28
13	3.43 ± 0.09 ^&^	0.38 ± 0.04 ^&^	27.57 ± 0.07 ^&^	31.15 ± 0.02 ^&^	36.37 ± 0.19 ^&^	95.09 ± 0.15 ^&^	1.17
14	4.55 ± 0.17 ^&^	0.52 ± 0.07 ^&^	25.27 ± 0.26 ^&^	25.62 ± 0.10	43.07 ± 0.36 ^&^	93.97 ± 0.03 ^&^	1.68
15	5.02 ± 0.11 ^&^	0.43 ± 0.02 ^&^	28.43 ± 0.19 ^&^	33.23 ± 0.08 ^&^	32.09 ± 0.21 ^&^	93.75 ± 0.12 ^&^	0.97
16	5.57 ± 0.02 ^&^	0.66 ± 0.02 ^&^	22.10 ± 0.22 ^&^	30.81 ± 0.03 ^&^	39.99 ± 0.16	92.90 ± 0.11 ^&^	1.30
17	4.34 ± 0.07 ^&^	0.53 ± 0.02 ^&^	22.72 ± 0.10 ^&^	29.02 ± 0.07 ^&^	42.42 ± 0.11 ^&^	94.16 ± 0.21 ^&^	1.46
18	4.96 ± 0.14 ^&^	0.49 ± 0.03 ^&^	18.77 ± 0.14 ^&^	29.72 ± 0.09 ^&^	45.01 ± 0.05 ^&^	93.50 ± 0.02 ^&^	1.51
19	5.19 ± 0.08 ^&^	0.91 ± 0.03 ^&^	26.64 ± 0.08 ^&^	26.05 ± 0.04	40.46 ± 0.01	93.15 ± 0.04 ^&^	1.55
20	4.47 ± 0.06 ^&^	0.43 ± 0.03 ^&^	23.74 ± 0.09	28.80 ± 0.10 ^&^	41.45 ± 0.19	93.99 ± 0.07 ^&^	1.44
21	4.17 ± 0.13 ^&^	0.78 ± 0.03 ^&^	23.91 ± 0.17	22.76 ± 0.16 ^&^	47.56 ± 0.18 ^&^	94.23 ± 0.31 ^&^	2.09
22	4.88 ± 0.09 ^&^	0.95 ± 0.02 ^&^	22.19 ± 0.30 ^&^	26.10 ± 0.08	44.95 ± 0.28 ^&^	93.24 ± 0.16 ^&^	1.72
23	3.84 ± 0.04 ^&^	0.68 ± 0.02 ^&^	24.14 ± 0.18	21.33 ± 0.02 ^&^	49.21 ± 0.17 ^&^	94.68 ± 0.15 ^&^	2.31
24	4.57 ± 0.11 ^&^	0.50 ± 0.03 ^&^	22.04 ± 0.18 ^&^	28.73 ± 0.13 ^&^	42.84 ± 0.16 ^&^	93.61 ± 0.15 ^&^	1.49
**25**	**5.34 ± 0.42 ^&^**	**0.51 ± 0.17 ^&^**	**16.21 ± 0.82 ^&^**	**31.61 ± 0.40 ^&^**	**45.23 ± 1.01 ^&^**	**93.06 ± 0.60 ^&^**	**1.43**
26	4.43 ± 0.06 ^&^	0.70 ± 0.03 ^&^	26.14 ± 0.12 ^&^	27.93 ± 0.04 ^&^	39.99 ± 0.23	94.06 ± 0.10 ^&^	1.43
27	5.03 ± 0.07 ^&^	0.40 ± 0.02 ^&^	20.01 ± 0.09 ^&^	23.19 ± 0.13 ^&^	50.73 ± 0.17 ^&^	93.93 ± 0.08 ^&^	2.19
**28**	**3.70 ± 0.05 ^&^**	**1.08 ± 0.05 ^&^**	**21.60 ± 0.13 ^&^**	**20.36 ± 0.09 ^&^**	**52.55 ± 0.12 ^&^**	**94.50 ± 0.06 ^&^**	**2.58**
**29**	**3.64 ± 0.16 ^&^**	**0.32 ± 0.03 ^&^**	**27.46 ± 0.03 ^&^**	**31.85 ± 0.08 ^&^**	**35.95 ± 0.14 ^&^**	**95.26 ± 0.25 ^&^**	**1.13**
30	5.21 ± 0.11 ^&^	0.81 ± 0.04 ^&^	22.06 ± 0.14 ^&^	29.11 ± 0.13 ^&^	41.96 ± 0.23 ^&^	93.14 ± 0.04 ^&^	1.44
31	4.92 ± 0.02 ^&^	0.45 ± 0.01 ^&^	29.44 ± 0.18 ^&^	34.96 ± 0.10 ^&^	29.26 ± 0.29 ^&^	93.66 ± 0.14 ^&^	0.84
32	4.05 ± 0.03 ^&^	0.68 ± 0.01 ^&^	22.41 ± 0.09 ^&^	28.11 ± 0.10 ^&^	43.80 ± 0.31 ^&^	94.32 ± 0.14 ^&^	1.56
33	4.46 ± 0.10 ^&^	0.76 ± 0.08 ^&^	20.52 ± 0.37 ^&^	23.84 ± 0.22 ^&^	49.44 ± 0.63 ^&^	93.80 ± 0.06 ^&^	2.07
34	4.88 ± 0.11 ^&^	0.64 ± 0.02 ^&^	26.18 ± 0.07 ^&^	20.23 ± 0.12 ^&^	47.09 ± 0.13 ^&^	93.50 ± 0.03 ^&^	2.33
35	5.06 ± 0.18 ^&^	0.64 ± 0.02 ^&^	24.62 ± 0.25 ^&^	30.73 ± 0.03 ^&^	37.97 ± 0.31 ^&^	93.32 ± 0.04 ^&^	1.24
36	3.93 ± 0.16 ^&^	0.65 ± 0.06 ^&^	24.62 ± 0.15 ^&^	32.68 ± 0.06 ^&^	37.10 ± 0.07 ^&^	94.40 ± 0.13 ^&^	1.14
37	3.82 ± 0.30 ^&^	0.86 ± 0.16 ^&^	21.47 ± 0.22 ^&^	31.01 ± 0.07 ^&^	41.67 ± 0.08 ^&^	94.14 ± 0.12 ^&^	1.34
38	4.18 ± 0.10 ^&^	0.59 ± 0.05 ^&^	23.12 ± 0.21	27.42 ± 0.14 ^&^	43.54 ± 0.30 ^&^	94.09 ± 0.13 ^&^	1.59
39	4.77 ± 0.13 ^&^	0.54 ± 0.01 ^&^	25.81 ± 0.22 ^&^	27.30 ± 0.10 ^&^	40.46 ± 0.26	93.58 ± 0.06 ^&^	1.48
40	4.48 ± 0.10 ^&^	0.68 ± 0.02 ^&^	27.25 ± 0.12 ^&^	30.89 ± 0.07 ^&^	35.93 ± 0.24 ^&^	94.06 ± 0.10 ^&^	1.16
41	4.64 ± 0.08 ^&^	0.64 ± 0.03 ^&^	25.75 ± 0.02 ^&^	32.98 ± 0.02 ^&^	35.23 ± 0.13 ^&^	93.97 ± 0.14 ^&^	1.07
42	3.53 ± 0.18 ^&^	0.43 ± 0.05 ^&^	25.72 ± 0.79 ^&^	24.90 ± 0.15	44.43 ± 0.81 ^&^	95.05 ± 0.18 ^&^	1.78
43	4.79 ± 0.12 ^&^	0.85 ± 0.01 ^&^	23.79 ± 0.18	23.70 ± 0.83 ^&^	46.04 ± 0.75 ^&^	93.53 ± 0.06 ^&^	1.94
44	6.11 ± 0.19 ^&^	0.75 ± 0.02 ^&^	25.37 ± 0.31 ^&^	34.02 ± 0.10 ^&^	32.72 ± 0.53 ^&^	92.11 ± 0.23 ^&^	0.96
45	4.44 ± 0.04 ^&^	0.38 ± 0.02 ^&^	25.58 ± 0.20 ^&^	28.44 ± 0.64 ^&^	40.58 ± 0.71	94.60 ± 0.12 ^&^	1.43
46	4.79 ± 0.17 ^&^	0.86 ± 0.03 ^&^	27.11 ± 0.14 ^&^	25.97 ± 0.05	40.51 ± 0.09	93.59 ± 0.09 ^&^	1.56
47	4.05 ± 0.12 ^&^	0.31 ± 0.02 ^&^	22.82 ± 0.13	36.75 ± 0.41 ^&^	34.99 ± 0.28 ^&^	94.56 ± 0.23 ^&^	0.95
48	4.65 ± 0.09 ^&^	0.50 ± 0.01 ^&^	25.61 ± 0.54 ^&^	25.22 ± 0.11	43.08 ± 0.41 ^&^	93.91 ± 0.13 ^&^	1.71
49	4.95 ± 0.11 ^&^	0.60 ± 0.01 ^&^	26.56 ± 0.22 ^&^	36.80 ± 0.67 ^&^	30.07 ± 0.80 ^&^	93.42 ± 0.10 ^&^	0.82
50	5.10 ± 0.06 ^&^	0.65 ± 0.02 ^&^	29.78 ± 0.20 ^&^	21.93 ± 1.01 ^&^	41.51 ± 0.92	93.22 ± 0.15 ^&^	1.89
51	5.23 ± 0.05 ^&^	0.60 ± 0.02 ^&^	23.90 ± 0.58	29.41 ± 0.29 ^&^	40.21 ± 0.65	93.52 ± 0.09 ^&^	1.37
52	5.54 ± 0.16 ^&^	0.61 ± 0.02 ^&^	22.54 ± 0.13	35.58 ± 0.87 ^&^	34.91 ± 1.08 ^&^	93.03 ± 0.19 ^&^	0.98
53	3.96 ± 0.16 ^&^	0.42 ± 0.02 ^&^	24.54 ± 0.26	28.96 ± 0.27 ^&^	41.41 ± 0.48	94.91 ± 0.17 ^&^	1.43
54	5.22 ± 0.03 ^&^	0.63 ± 0.02 ^&^	22.66 ± 0.26	27.06 ± 0.33	43.53 ± 0.47 ^&^	93.24 ± 0.09 ^&^	1.61
55	5.14 ± 0.12 ^&^	0.73 ± 0.02 ^&^	21.86 ± 0.27 ^&^	32.96 ± 0.31 ^&^	38.26 ± 0.63	93.08 ± 0.19 ^&^	1.16
56	5.05 ± 0.03 ^&^	0.64 ± 0.03 ^&^	24.50 ± 0.43	26.56 ± 0.82	41.96 ± 0.31	93.02 ± 0.32 ^&^	1.58
57	5.39 ± 0.03 ^&^	0.84 ± 0.02 ^&^	25.85 ± 0.70 ^&^	29.04 ± 0.32 ^&^	38.13 ± 1.05	93.02 ± 0.10 ^&^	1.31
58	4.93 ± 0.14 ^&^	0.75 ± 0.04 ^&^	26.90 ± 0.30 ^&^	25.78 ± 0.64	40.40 ± 0.89	93.09 ± 0.09 ^&^	1.57
59	4.43 ± 0.11 ^&^	0.61 ± 0.01 ^&^	23.16 ± 0.05	24.23 ± 0.50	46.41 ± 0.67 ^&^	93.80 ± 0.15 ^&^	1.92
60	3.86 ± 0.06 ^&^	0.48 ± 0.02 ^&^	24.86 ± 0.49	19.86 ± 0.06 ^&^	50.14 ± 0.51 ^&^	94.86 ± 0.15 ^&^	2.53
61	5.31 ± 0.04 ^&^	0.38 ± 0.00 ^&^	24.85 ± 0.41	22.59 ± 0.81 ^&^	46.27 ± 1.11 ^&^	93.71 ± 0.01 ^&^	2.05
62	5.04 ± 0.08 ^&^	0.47 ± 0.03 ^&^	24.31 ± 0.26	21.31 ± 0.13 ^&^	47.98 ± 0.17 ^&^	93.60 ± 0.08 ^&^	2.25
63	4.27 ± 0.26 ^&^	0.39 ± 0.02 ^&^	25.39 ± 0.55 ^&^	27.47 ± 0.30	41.68 ± 0.20	94.54 ± 0.30 ^&^	1.52
**64**	**5.35 ± 0.02 ^&^**	**0.46 ± 0.04 ^&^**	**29.82 ± 0.29 ^&^**	**26.16 ± 1.13**	**37.18 ± 1.12 ^&^**	**93.16 ± 0.08 ^&^**	**1.42**
65	4.42 ± 0.08 ^&^	0.60 ± 0.03 ^&^	21.62 ± 0.62 ^&^	27.18 ± 0.16	44.69 ± 1.15 ^&^	93.49 ± 1.09 ^&^	1.64
66	4.72 ± 0.29 ^&^	0.54 ± 0.07 ^&^	22.53 ± 1.54	24.96 ± 2.71	46.44 ± 1.44 ^&^	93.93 ± 0.15 ^&^	1.86
67	4.46 ± 0.27 ^&^	0.41 ± 0.03 ^&^	20.78 ± 1.56 ^&^	25.17 ± 1.34	48.11 ± 0.96 ^&^	94.07 ± 0.51 ^&^	1.91
**68**	**7.14 ± 0.24**	**2.31 ± 0.06**	**23.54 ± 0.15**	**25.66 ± 0.60**	**39.79 ± 1.13**	**88.99 ± 0.47**	**1.55**
Min	3.43	0.31	16.21	19.24	28.02	88.99	0.74
Max	7.14	2.31	29.82	37.74	53.02	95.26	2.76
Mean	4.68	0.62	24.34	28.01	41.41	93.76	1.55
SD	0.65	0.27	2.55	4.53	5.40	0.87	0.44

* PA: Palmitic acid, SA: Stearic acid, OA: Oleic acid, LA: Linoleic acid, ALA: α-linolenic acid. ^&^ *p* < 0.001 with respect to No.68 sample (‘Fengdan’), bolded are the cultivars and data of concern.

**Table 3:** Character statistics of excellent cultivars (mean ± SD, n = 3).

No.	Y (g) *	X1	X2	X3 (mm)	X4 (mm)	X5 (g)	X6 (%)	X7 (%)	X8 (%)	X9 (%)	X10
46	337.22 ± 65.14	907.67 ± 16.29	100.33 ± 35.13	9.20 ± 0.20	8.13 ± 0.35	24.65 ± 0.27	53.94 ± 1.19	21.24 ± 1.00	40.51 ± 0.09	93.59 ± 0.09	1.56
62	134.36 ± 60.08	288.00 ± 131.43	27.33 ± 9.29	11.33 ± 0.12	9.47 ± 1.11	29.33 ± 0.33	46.40 ± 0.41	15.13 ± 0.13	47.98 ± 0.17	93.60 ± 0.08	2.25
33	132.44 ± 46.72	483.67 ± 203.30	31.00 ± 6.00	8.47 ± 0.32	7.10 ± 0.20	22.82 ± 0.15	58.47 ± 1.54	24.69 ± 0.82	49.44 ± 0.63	93.80 ± 0.06	2.07
39	143.56 ± 31.91	368.33 ± 46.92	52.67 ± 6.66	11.00 ± 0.61	8.20 ± 0.26	27.36 ± 0.45	51.45 ± 1.58	18.99 ± 0.49	40.46 ± 0.26	93.58 ± 0.06	1.48
68	196.3 ± 38.31	524.67 ± 130.42	23.00 ± 2.00	9.13 ± 0.45	8.33 ± 1.38	28.81 ± 5.04	54.50 ± 2.96	20.87 ± 0.26	39.79 ± 1.13	88.99 ± 0.47	1.55

* Y: Seed yield per plant, X1: Seed number per plant, X2: Number of fruits per plant, X3: Seed length, X4: Seed width, X5: Hundred seed weight, X6: Kernel contents, X7: Oil contents, X8: ALA content, X9: Total UFA content, X10: n-3/n-6.

## Data Availability

The data presented in this study are available on request from the corresponding author. The data are not publicly available due to restrictions privacy.

## Appendix A

**Table A1:** Sixty-eight tree peony cultivars used in this study.

No.	Cultivars	No.	Cultivars	No.	Cultivars
1	‘Jinguimantang’	24	‘Chunlian’	47	‘Gejin’
2	‘Hongxia’	25	‘Jianshifen’	48	‘Mingmou’
3	‘Jinpaohong’	26	‘Baihuacongxiao’	49	‘Xianemao’
4	‘Sijinpaohong’	27	‘Cengzhongxiao’	50	‘Xiongmao’
5	‘Sihonglian’	28	‘Baihuazhancui’	51	‘Liming’
6	‘Hongxiuqiu’	29	‘Qinglongyaojinmo’	52	‘Hepingfen’
7	‘Pinghuqiuyue’	30	‘Mochijinhui’	53	‘Qingchun’
8	‘Junyanhong’	31	‘Xiuqunfang’	54	‘Renmiantaohua’
9	‘Chunzao’	32	‘Manyuanchunguang’	55	‘Mudanyan’
10	‘Honghe’	33	‘Xianchizhenghui’	56	‘Nongmozhongcai’
11	‘Chunhong’	34	‘Tingtingyuli’	57	‘Hongloucangjiao’
12	‘Yinhonglvbo’	35	‘Zhongqiuyueguang’	58	‘Hongyangfei’
13	‘Nichangyuyi’	36	‘Xueyuanhongxing’	59	‘Daonaiteng’
14	‘Hongxiazhenghui’	37	‘Hongbanbai’	60	‘Daodachen’
15	‘Baiyuanhongxia’	38	‘Ruhuasiyu’	61	‘Huadachen’
16	‘Xiaotaohong’	39	‘Yupantuojin’	62	‘Changshoule’
17	‘Fenyupan’	40	‘Xianhe’	63	‘Zihongdian’
18	‘Tianranfen’	41	‘Hehuafeicui’	64	‘Hongguishizi’
19	‘Yinbianmeigui’	42	‘Ziyun’	65	‘Fangji’
20	‘Chunsemanyuan’	43	‘Zilanpan’	66	‘Xugang’
21	‘Haibo’	44	‘Jiali’	67	‘Baishen’
22	‘Caiyelanju’	45	‘Liuyehong’	68	‘Fengdan’
23	‘Hudiebaochun’	46	‘Zibanbai’		

**Table A2:** The agronomic characters of 68 tree peony cultivars (mean ± SD, n = 3).

No.	Y (g) *	X1	X2	X3 (mm)	X4 (mm)	X5 (g)
1	32.18 ± 3.85	125.33 ± 20.5	27.00 ± 2.00	8.37 ± 1.12	7.37 ± 0.81	16.31 ± 0.84
2	36.36 ± 14.67	99.33 ± 44.55	48.33 ± 8.14	9.33 ± 0.84	8.43 ± 0.31	25.34 ± 1.13
3	56.62 ± 28.99	218.33 ± 101.4	39.67 ± 19.14	8.60 ± 0.30	7.17 ± 0.60	15.33 ± 0.79
4	32.71 ± 14.26	112.67 ± 45.08	26.33 ± 5.13	9.13 ± 0.31	7.87 ± 0.60	19.31 ± 0.51
5	42.08 ± 23.73	102.33 ± 61.04	38.33 ± 13.32	10.13 ± 1.16	7.90 ± 0.44	28.51 ± 1.64
6	34.79 ± 15.28	107.33 ± 38.28	43.33 ± 15.63	10.87 ± 0.74	8.07 ± 0.31	23.30 ± 1.35
7	54.59 ± 46.92	203.67 ± 173.18	65.67 ± 46.72	9.17 ± 0.92	7.33 ± 0.76	21.13 ± 1.15
8	48.68 ± 5.04	148.67 ± 7.02	33.00 ± 7.00	9.07 ± 0.47	7.13 ± 0.59	23.88 ± 0.63
9	34.72 ± 16.19	201.00 ± 109.38	26.33 ± 8.39	8.67 ± 1.12	6.90 ± 0.50	12.93 ± 1.08
10	31.22 ± 16.09	111.00 ± 63.66	44.00 ± 8.00	10.00 ± 0.50	6.70 ± 0.36	18.01 ± 0.43
11	39.05 ± 21.10	121.67 ± 58.45	26.33 ± 6.66	8.93 ± 0.97	7.83 ± 0.60	22.82 ± 0.45
12	42.41 ± 16.16	231.00 ± 86.09	48.00 ± 15.10	7.83 ± 0.23	7.03 ± 0.15	15.08 ± 0.20
13	35.62 ± 18.00	123.00 ± 63.17	37.00 ± 2.00	9.43 ± 0.32	8.17 ± 1.17	23.26 ± 0.70
14	33.38 ± 10.75	122.67 ± 22.68	27.33 ± 15.04	8.60 ± 1.06	7.37 ± 0.31	19.91 ± 1.30
15	60.66 ± 2.95	241.33 ± 8.96	41.67 ± 13.50	9.77 ± 0.15	6.70 ± 0.62	17.28 ± 0.89
16	31.69 ± 15.72	124.00 ± 96.60	39.33 ± 1.53	7.83 ± 0.40	7.10 ± 0.44	15.11 ± 0.53
17	56.38 ± 16.55	150.00 ± 40.36	35.67 ± 11.15	9.33 ± 0.06	8.00 ± 0.87	25.46 ± 0.34
18	35.43 ± 20.73	100.67 ± 55.59	15.33 ± 7.02	9.37 ± 0.31	8.27 ± 0.85	26.31 ± 0.82
19	46.50 ± 4.11	131.67 ± 15.01	46.50 ± 4.50	9.47 ± 0.40	7.13 ± 0.25	22.03 ± 0.04
20	43.51 ± 11.40	134.67 ± 30.01	14.33 ± 3.21	9.17 ± 0.55	7.83 ± 0.57	24.43 ± 0.79
21	47.29 ± 11.49	175.33 ± 38.5	25.00 ± 5.20	8.83 ± 0.50	7.33 ± 0.72	20.59 ± 0.63
22	126.96 ± 13.93	421.33 ± 54.15	43.67 ± 5.03	9.00 ± 0.30	7.30 ± 0.26	21.52 ± 1.27
23	35.66 ± 0.84	128.00 ± 10.00	34.50 ± 3.50	9.13 ± 0.61	7.93 ± 0.65	21.44 ± 0.27
24	38.51 ± 17.02	145.00 ± 72.02	13.00 ± 4.58	8.53 ± 0.06	7.60 ± 0.36	22.22 ± 0.47
25	85.78 ± 11.99	326.67 ± 37.00	37.00 ± 3.46	8.07 ± 0.12	7.13 ± 0.29	19.82 ± 0.74
26	38.85 ± 5.76	127.33 ± 13.05	41.67 ± 9.50	10.57 ± 0.42	8.47 ± 0.21	25.35 ± 0.73
27	62.32 ± 15.74	237.33 ± 69.95	73.67 ± 31.50	9.17 ± 0.38	7.83 ± 0.58	18.18 ± 1.32
28	74.35 ± 83.82	169.67 ± 191.72	69.00 ± 66.57	10.13 ± 0.35	8.70 ± 0.56	28.85 ± 0.62
29	76.13 ± 27.41	334.67 ± 72.97	44.67 ± 8.96	8.43 ± 0.35	7.67 ± 0.55	17.34 ± 0.27
30	31.96 ± 1.05	121.67 ± 1.53	27.00 ± 7.00	9.97 ± 0.25	8.10 ± 0.82	28.25 ± 0.58
31	31.66 ± 21.70	91.33 ± 56.15	30.33 ± 2.89	10.87 ± 0.21	8.77 ± 0.81	26.59 ± 0.99
32	59.26 ± 12.78	206.00 ± 42.14	44.33 ± 5.51	9.03 ± 0.06	7.20 ± 1.04	24.53 ± 0.26
33	132.44 ± 46.72	483.67 ± 203.30	31.00 ± 6.00	8.47 ± 0.32	7.10 ± 0.20	22.82 ± 0.15
34	54.85 ± 34.22	151.33 ± 79.35	31.00 ± 15.72	10.73 ± 1.10	7.87 ± 0.15	25.53 ± 0.13
35	33.25 ± 6.87	118.67 ± 36.23	21.33 ± 4.51	8.97 ± 0.80	7.77 ± 0.15	16.36 ± 0.49
36	126.81 ± 45.05	462.33 ± 177.73	25.00 ± 12.77	8.50 ± 0.50	6.77 ± 0.51	21.55 ± 0.29
37	84.93 ± 31.65	260.33 ± 80.43	39.33 ± 12.50	10.90 ± 0.78	8.17 ± 0.46	23.82 ± 0.90
38	119.88 ± 21.24	451.67 ± 61.16	54.00 ± 5.29	9.53 ± 0.23	8.17 ± 0.15	20.52 ± 0.84
39	143.56 ± 31.91	368.33 ± 46.92	52.67 ± 6.66	11.00 ± 0.61	8.20 ± 0.26	27.36 ± 0.45
40	69.46 ± 17.38	280.67 ± 150.63	37.33 ± 8.62	7.63 ± 0.06	7.33 ± 0.06	21.16 ± 0.82
41	69.46 ± 3.08	236.33 ± 14.64	26.67 ± 4.16	10.20 ± 0.36	7.57 ± 0.40	21.49 ± 0.43
42	39.78 ± 8.44	110.00 ± 28.16	44.67 ± 6.81	9.47 ± 0.06	8.93 ± 0.47	28.09 ± 0.82
43	67.45 ± 6.82	191.67 ± 28.75	39.67 ± 6.66	9.33 ± 0.21	8.37 ± 0.38	24.69 ± 0.22
44	53.17 ± 17.34	272.33 ± 86.94	32.33 ± 11.59	8.90 ± 2.16	6.70 ± 0.52	17.65 ± 0.22
45	35.26 ± 34.06	83.33 ± 71.47	24.67 ± 14.84	9.40 ± 0.26	8.30 ± 0.66	19.64 ± 0.12
46	337.22 ± 65.14	907.67 ± 16.29	100.33 ± 35.13	9.20 ± 0.20	8.13 ± 0.35	24.65 ± 0.27
47	34.66 ± 13.23	130.00 ± 52.12	27.00 ± 6.24	9.10 ± 0.46	7.27 ± 1.01	18.95 ± 0.27
48	57.03 ± 30.68	318.00 ± 156.49	25.67 ± 11.02	7.40 ± 0.46	6.87 ± 0.21	17.57 ± 0.19
49	33.99 ± 29.20	172.00 ± 144.04	30.00 ± 16.70	11.50 ± 1.40	7.90 ± 0.46	18.99 ± 0.43
50	42.44 ± 3.82	314.00 ± 37.72	29.00 ± 5.29	8.33 ± 0.06	6.80 ± 0.26	13.19 ± 0.06
51	44.55 ± 15.85	220.00 ± 104.22	30.67 ± 18.50	8.97 ± 0.15	6.63 ± 0.90	18.63 ± 0.47
52	50.29 ± 41.74	270.00 ± 212.06	27.00 ± 17.58	8.63 ± 0.15	7.40 ± 0.85	17.42 ± 0.37
53	71.91 ± 37.81	425.00 ± 222.58	43.33 ± 9.29	9.53 ± 0.06	5.83 ± 0.64	16.30 ± 0.36
54	57.29 ± 57.59	214.67 ± 189.58	18.00 ± 2.00	9.73 ± 0.06	7.63 ± 0.59	21.93 ± 0.04
55	99.62 ± 67.45	347.33 ± 217.98	40.00 ± 12.49	11.47 ± 1.25	7.83 ± 0.55	23.42 ± 0.22
56	34.65 ± 19.79	149.00 ± 90.15	78.00 ± 40.95	10.77 ± 0.23	7.37 ± 0.57	22.16 ± 1.44
57	70.42 ± 55.68	292.33 ± 226.6	77.33 ± 25.81	9.20 ± 0.62	8.23 ± 0.46	23.41 ± 0.29
58	38.15 ± 27.94	212.33 ± 164.49	22.00 ± 15.10	9.53 ± 0.47	7.03 ± 0.85	17.43 ± 0.44
59	97.27 ± 47.94	465.33 ± 188.88	54.00 ± 14.80	9.27 ± 0.45	7.80 ± 0.78	19.55 ± 0.46
60	42.96 ± 10.26	134.33 ± 47.09	31.00 ± 16.82	10.37 ± 0.38	7.47 ± 0.95	23.28 ± 0.14
61	35.76 ± 27.82	112.33 ± 83.76	20.00 ± 2.65	10.57 ± 0.12	7.87 ± 0.80	21.92 ± 2.02
62	134.36 ± 60.08	288.00 ± 131.43	27.33 ± 9.29	11.33 ± 0.12	9.47 ± 1.11	29.33 ± 0.33
63	51.69 ± 3.63	321.00 ± 40.78	44.67 ± 3.79	7.47 ± 0.42	6.33 ± 0.21	13.47 ± 0.37
64	34.95 ± 3.88	157.00 ± 39.13	31.33 ± 8.14	10.80 ± 0.72	8.37 ± 0.55	23.34 ± 3.13
65	32.64 ± 37.00	107.33 ± 116.23	26.00 ± 9.54	9.37 ± 0.32	7.63 ± 0.65	23.54 ± 0.16
66	41.77 ± 22.51	152.00 ± 75.39	54.67 ± 18.50	9.13 ± 0.15	8.47 ± 0.68	23.68 ± 0.70
67	59.23 ± 12.18	251.67 ± 48.00	15.00 ± 3.61	8.67 ± 0.59	8.07 ± 0.25	15.31 ± 0.30
68	196.3 ± 38.31	524.67 ± 130.42	23.00 ± 2.00	9.13 ± 0.45	8.33 ± 1.38	28.81 ± 5.04

* Seed yield per plant (Y), Seed number per plant (X1), Number of fruits per plant (X2), Seed length (X3), Seed width (X4), hundred seed weight (X5).
